# The MAP (Me-As-a-Process) coaching model: a framework for coaching women’s identity work in voluntary career transitions

**DOI:** 10.3389/fpsyg.2024.1364134

**Published:** 2024-05-30

**Authors:** Sarah Snape

**Affiliations:** Independent Practitioner, London, United Kingdom

**Keywords:** identity work, identity, gender, career transition, coaching

## Abstract

Dealing with change and the resulting process of transition is challenging. In today’s workplace, where change and innovation are increasingly a fact of life, too many transitions end in failure, at a high cost to both people and organizations. Interest in the identity work integral to career transition has grown rapidly in recent years and it is now recognized that career transition is more than simply a change in status, salary and role description. It involves social, relational and personal shifts, conscious and unconscious processes, and identity work—agentic, holistic engagement in the shaping and sustaining of who we become. Evidence suggests that specifically addressing identity work in coaching leaders, teams and groups significantly increases the success rate of transitions. And yet topics around identity and identity work are given little prominence in coaching education, leaving many coaches unaware of these basic constructs. This paper presents a new coaching framework, the MAP (Me-As-a-Process) coaching model, to support coaches and their clients as they embark on the process of identity work in voluntary career choices and transitions. It draws on research from my qualitative doctoral study (2021) which identified four stages in the process of women’s identity work in voluntary career change and choice. It synthesizes academic theory, evidence from coaching practice, and findings from 53 women who had recently experienced career choice or change.

## Introduction

As an executive coach, I find that career choices and transitions are a staple feature of coaching sessions (Yates et al., 2017) and involve identity and identity work (Ashforth and Schinoff, 2016; Ibarra and Obodaru, 2016). Here in the West, what we do for a living has increasingly become a primary method of self-definition. Many of us find identity in, and are identified by, the work we do (Baumeister and Muraven, 1996; Gini, 2001; Ibarra, 2003). It is therefore not surprising that people can find the experience of career transition challenging. Work-related change involves a shift in identity (Ibarra, 1999) and requires identity work (Petriglieri et al., 2018). Identity work involves activities by which individuals consciously form, repair, reject, or revise their public selves and their personal identity to create a congruent sense of self (Alvesson and Willmott, 2002; Ashforth et al., 2008; Brown, 2015).

The very nature of careers, organizations and work are all undergoing significant conceptual shifts in terms of meaning and role in our society, with a move toward more mobile, flexible and plural working lives (Brown, 2015, 2017; Parker, 2016; Caza et al., 2018). Identity work in career transitions plays an increasingly significant role in sustaining a sense of personal agency, continuity, coherence and self-esteem (Petriglieri and Petriglieri, 2010). Yet “*how* work-related identity change occurs remains largely under-studied and poorly understood” (Ibarra, 2005, p. 1; Fouad and Bynner, 2008; Petriglieri et al., 2018).

My clients are often at points of voluntary transition: a promotion; a move to a new organization; a re-alignment of attitude toward an existing role; a sideways move into a different area of the business; a child-or maternity-related decision; a career change or break; or an offer of voluntary redundancy. They find themselves struggling to adapt to their situation. They might express their difficulties in language such as “I do not know who I am meant to be”; “should I be acting differently?”; “I do not feel I fit in,” “is this right for me?” and “what are people thinking/saying about me?” These issues relate to their sense of identity, their subjective interpretation of how they are seen, and how they see themselves (Gecas, 1982; Baumeister and Muraven, 1996; Brewer and Pickett, 1999).

However, executive coaching has tended to address the concept of identity obscurely, as part of exploring personal leadership styles, for example, or in one-to-one coaching programs focused on behavioral change. Participants have noted retrospectively that coaching did indeed help with identity construction, but without specifically tackling it or naming it (Bonneywell, 2016). In her research on the link between identity and coaching practice, Butcher (2012, p. 123) noted that “concepts around identity were integral to the coaching process and dynamic.” Yet she also noted that executive coaches can be reluctant to address identity, proposing that this may be related to concern about professional boundaries and beliefs that “identity” comes into the realm of therapy rather than coaching. Brown (2017) proposes that under-specification of “identity” in organizational research dealing with identity-related issues has limited its usefulness in practice.

This reluctance to address the process of identity work is perhaps at least partly a result of the disinclination to include identity-related issues in coaching education (Evans and Lines, 2014; McGill et al., 2019). While the 21st century has seen a marked increase in research on career transitions (Caza et al., 2018) and a surge in coaching literature, a parallel increase specifically on coaching for transition and its associated identity-related issues has yet to emerge, “and presents itself as an opportunity for future research” (Parker, 2016, p. 419; McGill et al., 2019). It has been established that coaching for transition has a positive effect (Motulsky, 2010). However, until now coaching has tended to approach career transition from a skills and performance perspective which may be ineffective in achieving the desired results (Skinner, 2014; Vähäsantanen et al., 2017), rather than engaging with and supporting the whole person with the more fundamental, holistic issue of shifting identities (Parker, 2016; Hibbert et al., 2017).

Given this gap in the literature, and my experiences of coaching periods of choices and change in the workplace, I wanted to discover how this identity work was experienced, so as to make my coaching more effective. Ibarra’s (2003) *Working Identity* was my first port of call, and it was her work that made me realize that the area of identity and identity work in career transitions needed to be in a coach’s toolkit. So in 2018, I embarked on a doctoral study of women’s identity work in voluntary career choices and transitions. I limited the research to women partly because I am female, and seek to contribute research that supports other women effectively. Also, while I work with both men and women and find that their experiences of identity work in career transitions are in many ways similar, I find that women’s experience is potentially richer as it is more complex and multi-layered (Motulsky, 2010; Mavin et al., 2014), encompassing as it does gender inequality in our society and in the workplace (Government Equalities Office, 2019; FTSE Women Leaders, 2024), and maternity issues.

Multiple studies have found that women struggle to work out who they are and what they want due to their complex and multiple roles, and therefore find it difficult to make career choices (Gersick and Kram, 2002; Ruderman and Ohlott, 2005). “Identity is about meaning and mattering” (Parker, 2016, p. 427), so focusing on identity during times of career transition is a key coaching discussion, encouraging clients to step back and take a holistic view of the self they want to actively craft (Watson, 2008). Theories such as “identity as adaptation” (Baumeister and Muraven, 1996), multiple and shifting identities (Brewer and Pickett, 1999), possible and impossible selves (Markus and Nurius, 1986; Sturgess, 1999; Ibarra and Obodaru, 2016) and “woman as a project” (Savickas et al., 2009; Mavin et al., 2014) provide concepts for women to navigate in the safe space of a coaching session while thinking about their own experiences. Skinner (2014, p. 110) describes the coach as an “enabler of identity formation.”

I have found that introducing discussions around concepts of identity can encourage a sense of objectivity that inspires women to explore their pasts, presents and futures in a dispassionate way. My clients often report an increased sense of empowerment and confidence in who they are, what they do and how they behave, and a greater awareness of the choices they are making.

## The existing literature

I wanted to gain an understanding of how to coach the process of women’s identity work in career choices and transitions most effectively, as this was an issue I found myself increasingly facing in my coaching practice. There was little to guide me in the existing literature. The individual components of the topic have attracted many researchers, but no-one has yet collated their findings within a coaching study. Ibarra’s *Working Identities* (Ibarra, 2003) was the closest I could find, but it was not centered on women, nor aimed at coaching or coaches. Nonetheless, her stories were directly relevant to my experiences in coaching practice and proved a good launching pad. Initial research was directed by looking deeper into the ideas she described and consulting her bibliography. From there, other key sources were examined, and a picture built of the broader existing theoretical landscape. The research strategy was to first become familiar with work published on each of the individual key concepts, and then to search for coaching literature in these areas. Consequently, an extensive literature review focused on four overarching areas: identity and identity work in career transitions; career transitions; gender in career transitions; and coaching women’s identity work in career transitions. The review was broad and drew on literature from a range of disciplines including psychology, gender studies, executive coaching and career development.

An understanding of “identity” was informed by researching its development through history, from Erikson (1959) and Gecas (1982) through to Petriglieri et al. (2018). Contemporary views of identity describe it as a socially constructed, contextual, malleable, multiple concept over which individuals have a degree of agency (Skinner, 2014; Yates, 2017a). Interest in the construct of identity has increased and it has become a popular lens through which to investigate phenomena (Alvesson et al., 2008). For the purposes of this study, Rodgers and Scott’s definition of identity (Rodgers and Scott, 2008, p. 733) was the one I preferred:

“(1) that identity is dependent upon and formed within multiple contexts which bring social, cultural, political, and historical forces to bear upon that formation; (2) that identity is formed in relationship with others and involves emotions; (3) that identity is shifting, unstable and multiple; and (4) that identity involves the construction and reconstruction of meaning through stories over time.”

There has been an explosion of research on “identity work” since Snow and Anderson first brought it to attention in 1987 (Caza et al., 2018). There is consensus that identity work (also sometimes referred to as identity construction) refers to the interlinked activities by which individuals consciously or unconsciously create, maintain, repair, display, revise and discard social, personal and professional identities (Brown, 2017). Identity work is not a one-off accomplishment, nor is it done in isolation; rather it is an ongoing, conscious negotiation with recognizable stages and activities (Petriglieri and Petriglieri, 2010; Sinclair, 2011; Savickas, 2012; Evans and Lines, 2014).

In contemporary understanding, “career” describes a series of work-related choices that individuals make over a life span (Fouad and Bynner, 2008). Careers today are characterized by change. They require ongoing revisions of our personal definitions of success, often entailing periods of questioning, exploring, testing and reorientation (Petriglieri et al., 2018). These revisions and reinventions are not limited to competencies, attitudes and behaviors, but also include a reorganization of our basic priorities and organizing principles—our “working identity” (Ibarra, 2003). Baumeister and Muraven (1996) argue that the diminished role of religion in the Western world as provider of a value base for everyday choices has led to the elevation of the self to fill that gap, with work becoming a primary source of meaning. Work is our mark of identity, our signature on the world; it defines us, and shapes our view of the world (Gini, 2001).

“Career transitions” can involve a task change, a position change, or an occupation change encompassing a completely new role and set of tasks (Heppner, 1998). Alternatively, they may involve a change in orientation toward a role already held (Louis, 1980). Scholars argue that an internal psychological transition accompanies the external change (Bridges, 1980; Ibarra, 2003; Parker, 2016). Periods of uncertainty and self-doubt, of not-knowing, in which we are stimulated to stabilize our self-conceptions by constructing our identities (Fachin and Davel, 2015), accompany the search for an answer to the holistic question “How can I become who I want to be?” (Yates, 2017a, p. 4). Transitions present challenges to our self-understanding, identity threats, and involve not only the sustaining of “who I am” but also the search for and discovery of “who I want to become” (Fachin and Davel, 2015). The process of transition is often understood as a three-stage process: the end of one state, followed by an in-between period of transition often referred to as “liminality” (Van Gennep, 1960), then a new beginning; all stages being of indeterminate length and activities. The middle stage, liminality, involves an identity struggle between the old and the new, a loss of the familiar before a familiarity is established with the new, a clash between the attributes and behaviors required in “who I have been in the past” and “who I will become.” Coping with this change involves the person, the environment and the complex relationship between them (Schlossberg, 1981).

Existing studies find that to be effective, coaching for transition must be highly individualized (Leimon et al., 2011), as the psychological impact of the change does not necessarily correlate to its magnitude. Coaching that takes a whole-life approach, focusing on identity rather than career, has a significant positive impact on job success and satisfaction (Skinner, 2014; Yates, 2017a). Coaching for transition can encourage a new sense of self (Pinkavova, 2010) and can encourage women to experience growth and development at career turning points, enabling them to embrace new challenges (Motulsky, 2010; Skinner, 2014).

While individual elements of the topic have inspired research studies, there is a gap in coaching literature relating specifically to coaching the process of women’s identity work in career choices and transitions. This study maps the crafting of revised identities as women experienced discontinuous careers and engaged in “struggles between competing conceptions of what a good career and life should entail” (LaPointe, 2013, p. 142), in order to raise coaches’ awareness and enable them to support women more effectively.

## The research: methodology and scope

The goal of the research was to support coaching practice by creating a model that could map women’s experience of identity work in career choices and transitions. I wanted to create a bridge between the theoretical concepts involved in this topic and coaching in practice; to co-create, with working women, an accessible coaching model based on their experiences of identity work in career choices and transitions. This would involve discovering, testing, and generating.

Some critics of models agree with Burr (2015) that the act of attempting to encapsulate an experience in a conceptualization can be seen as a form of essentialism or reductionism, entrapping, labeling and disempowering people in an attempt to clean up the messiness and variance of human experience, capturing and freezing “a complex phenomenon in terms of simpler or more fundamental elements” (Burr, 2015, p. 7). However, others counter this by clarifying that conceptualizations can be a stimulus for an investigation, rather than a predictor or controller of behavior (de Rivera and Kreilkamp, 2006), serving as a provisional map of phenomena, rather than a definition of how things happen. “All that one can hope for is to gain some insights into the complex patterning that exists” write Guba and Lincoln (1988, cited in Fetterman, 1988, p. 95). As a coach, I have found that models facilitate conversations which clients may be otherwise unable or reluctant to articulate. They can help to bring to consciousness what may otherwise be a sub-conscious process (Bourne and Özbilgin, 2008), and represent the complex, concurrent experience of both wanting to be the same as others, and to be unique (Brewer, 2003): “*this bit reflects my experience, this bit does not*.” Models can provide a basic language for a description of what is happening for the client, as reported by Annabel, one of the research partners: “*It’s finding the language, it’s seeing these written down, and I’m like, oh my God that’s how I’m feeling. I think even if I did have people to talk to, I cannot find the language to explain what I’m really trying to get to*.” Annie, another research partner, felt a model “*helps organize your thoughts during transition – controls the chaos*”; while for Clara, they “*give a frame to this complexity and enormity of being me.*” Many other research partners felt validated by models, and a sense of relief: “*I’m not the only one going through this*.”

The methodology used to achieve the goal of this research reflects my own constructivist worldview, in which knowledge is never fixed but continually evolving in response to an iterative process of exposure to experiences, theories and understandings. The study was therefore designed to reflect these philosophical underpinnings. Current thinking on the various concepts embraced by this study has evolved over decades, and the literature review provided definitions and understandings that were continually refined as new evidence was gathered and theoretical revisions proposed. Identity work itself, at the very heart of this research, is generally understood to be a never-ending “ongoing process” (Savickas, 2012; Evans and Lines, 2014), rather than a one-off process. This idea of continually “becoming,” of creating the realities in which we participate (Charmaz, 2006), resonated closely with my constructivist worldview and extended to all aspects of this study.

An interpretative qualitative research approach was considered the most appropriate for this explorative, idiographic, highly personal study. Multiple subjective realities were explored using a methodology introduced by de Rivera in 1981: Conceptual Encounter (CE). This fits perfectly with a constructivist approach, inviting participants to become research partners in the co-creation of a conceptualization that maps the issue being explored. CE studies are scarce compared to those using other methodologies. While this limits the number of existing studies to draw on, it also presents an opportunity to tackle a research topic from a new perspective.

Conceptual Encounter methodology requires researcher involvement in the generation of an initial conceptualization based on practitioner experiences and literature research. This initial model is tentative and exploratory, and subject to ongoing revision as more input is received from research partners. The interviews begin with an exploration of a research partner’s experience of the topic in question. Data is recorded, to be analyzed after the interview. In the second half of the interview, the initial conceptualization is shown to a research partner or focus group, and they are invited to accept, reject or add to the elements of the model. After the interview, the researcher analyses the data and takes in modifications to the model, and then takes that modified model to the next research interview. In this way, the model iteratively evolves. This process of collecting, analyzing and interpreting the data and integrating experience continues until the model makes explicit what was previously implicit; achieves saturation; and is “elegant and parsimonious” (de Rivera and Kreilkamp, 2006, p. 8). In this way, the conceptualization generated by the researcher and the research partners becomes a genuine co-creation and is a synthesis of theory, practice and evidence. It is an emergent methodology, an iterative process, a negotiated consensus.

While the act of creating an initial model might be seen to contain elements of a deductive approach, in fact the CE approach is one of co-creation, where researcher and research participants collaborate on a model representing their experience. Both the researcher and the research partners contribute not only to the content of the research, but also to the creative thinking that generates, manages and draws conclusions from the research.

The evidence for this CE research emanated from primarily retrospective, subjective accounts of women’s identity work in career transitions. Research partners included 12 coaches (involved in the generation of the initial model) and 41 women age 25–55, who are working, or had been until recently, in organizations within multiple sectors across the UK. The women were at different stages in their transitions at the time of interview, but all were either experiencing a voluntary career transition or had done so within the last 2 years. Participants were representative of those liable to be offered executive coaching by their organizations—the sample was largely highly educated, relatively privileged, high earning, and relatively confident. Only 5 out of 53 participants were from ethnic minorities, which is a limitation of this study. However, “if a given experience is possible, it is also subject to universalization” (Haugh, 1987 cited in Willig, 2001, p. 17); and this research is just the beginning of a conversation and exploration, rather than prescriptive or predictive.

The study is limited to voluntary career transitions, generally accepted as referring to self-initiated work transitions driven by personal agency, as opposed to involuntary transitions shaped by external constraints (Fouad and Bynner, 2008). However, as the research progressed, some ambiguity and complexity emerged with this concept. Voluntary redundancy, for example, offered to some participants with a financial incentive, proved hard to resist but was later regretted. A senior promotion offered unexpectedly and without a normal application process, with a 24-h deadline for acceptance, led to doubts and questions later. Other cases involved difficulties with working situations, where change was not desired but participants felt they had been subtly or overtly pressured into taking action. However, whatever the circumstances prior to the transition, all of the participants reported that the final decision to act had been their choice and that they in no way saw themselves as victims. Rather, they felt empowered by their actions.

## Findings: women’s experiences of identity work in career choices and transitions

One of the most consistent findings from this group of research partners was that they appeared confused by what they meant by the terms “identity” and “identity work.” In a world in which the word “identity” has been popularized across multi-disciplinary areas (Brown, 2017), these research participants relished the opportunity offered by this research to name and explore it. Rather than aligning with the currently prevalent view of identity as holistic and multiple (Fachin and Davel, 2015), they tended to rely on their career for an explanation of who they were, as described by Ava: *Who am I, without being this role? Because it’s a sort of defining, people know me for that, that’s who I am.* Anastasia went further: *Career has always been at the center of my identity and everything else I build around that*.

The research interview included a brief introduction to the concept of identity as described in the literature and within that a discussion of multiple selves (Caza et al., 2018). The idea that a self was not one thing exclusively, such as “mother,” “career,” “carer,” but could embrace several identities which can co-exist, shift and be shifted to accommodate changing circumstances (Ashforth and Schinoff, 2016), was a relief to many of the research participants, as expressed by Annie: *45 min ago, I thought I was only my career, now I realize I’m not. A real relief*.

The concept of identity work was similarly unclear to most of the participants, but once explained, it was welcomed. Phrases referring obliquely to identity work occurred regularly throughout the interviews, such as *I had to do a lot of work on myself* (Alex) or *This idea of becoming more you … all of the experiences I’m having now will contribute to this amazing person* (Ada). The majority of the research partners had experienced the challenging nature of career transitions, and did not predict an easy ride. Butler (2005, p. 103) commented: “Becoming human is no simple task and it is not always clear when or if one arrives.” In this 2020 study, participant Angela echoes that sentiment in describing the ongoing, evolving project of “Becoming”: *It’s hard work, being a person.* The data showed a clear desire for personal responsibility and agency in careers and life, and a willingness to accept responsibility for making choices in their actions and reactions.

In evaluating and coding the data from the interviews, themes were allowed to emerge rather than being restricted by the existing threads from the initial and evolving model. This was to encourage fresh thinking that could challenge or support the initial and evolving model. The themes that emerged, however, related to the four stages of the model and are therefore presented under the headings of the four stages: “Who am I now?”; “Who could I become?”; “Who do I really want to be?”; and “How can I make that happen – and still be me?” Findings were also used to modify the conceptualization and to create the accompanying coaching questions.

The data was revisited many times in an ongoing, iterative process of evaluating and modifying, and thematic findings were distilled. Quotations are deliberately lengthy in order to provide context, and to give participants their voices.

**Stage one, Who am I now?,** represents an exploration of identity, current situation, expectations and assumptions and “what got them here.” This heightened self-awareness is recognized as a key early stage in the process of personal growth and expansion (Whitmore, 1992; Downey, 2003). Research partners reported this stage to be lengthy: “*It’s a bit like getting the ground tilled or plowed so that it’s ready to be able to receive a new idea*.” (Alison).

Prevalent in the data from this part of the interview was anger and overwhelm at the multiple and complex, covert and overt consequences of our gendered society. Without being specifically asked about it, almost all the women referred to the issue of gender, with emotional responses ranging from fury and frustration to resignation. Aria could not understand why she should be treated in any way differently to a man, as she did not want to have children: *I have always known I would never be a mother … I do not see that my identity should be any different from a man’s. I just do not get that…I am a person. I believe my gender has caused me problems in the past so I think my gender has affected my identity, but I am very angry about that.*

Gendered parental expectations were presented as influential, creating a push to confound or a pull to comply, often both. Anastasia described her parents’ reaction to her becoming a mother: *When child number one arrived, I discovered that my parents thought I should give up working and stay at home. Complete shock because I never in a million years thought that that was what their expectation was … The level playing ground that we all thought we were growing up in, turned out even though the parents were telling us, they did not believe it enough to think that that’s what happened when you actually had children.*

For many of the women, the simple fact of female biology, their ability to reproduce, was a fundamental issue, an underlying element in their view of their identity and their plans for the future. Amid the strong desire for autonomy to make their own life choices, they perceived gender-focused expectations as an area in which control and clarity were lacking. These included gendered parental expectations, the gendered culture at the top of organizations, exasperation with men’s inability to step up at home, and their own confusion about how they wanted to operate around the dual arenas of career and raising their children.

**The second stage, “Who could I become?”**, incorporates “identity play”—an enjoyable time of imagining, dreaming, exploring, as described by Amina: *“The world was my oyster; I could do anything … I feel like number two was a fairly long stage.”* This stage involves indulging in fantasies about possible or indeed impossible selves. For the participants, exploring possible selves (Markus and Nurius, 1986) was a relief from the stresses of working life. Some expressed fantasies of giving up a lucrative but stressful career in favor of a menial role, representing a desired identity in which the negative trappings of ambition are replaced by a simpler life choice. Others dreamed of more responsibility, or expanded horizons. Possible selves in this study included a pilot, an undertaker, a CEO, a full-time mum, a sky-diver and many others. Research partners enjoyed this phase of entertaining “possible selves” without having to subject those dreams to scrutiny or explore possible consequences, and recognized that they provided information relating to their current situation. This stage provides key information about what individuals really want, their “identity motives” (Vignoles et al., 2008). For this group of research partners, identity motives were Control and agency; Challenge and learning; Values and authenticity; Meaning and purpose; and Finding a balance. This stage of exploring possible selves also reinforced empowerment and control, as the women realized they had, and indeed were, exercising choice in how they lived their lives.

**In the third stage, “Who do I really want to be?”**, choices are narrowed down and subjected to closer examination of whether and how identity can be reoriented toward desired futures, from personal, social and practical perspectives. This may lead to a decision to make significant change, or to a choice to continue as before, but with a new purpose and attitude. Moving from the inward-looking, reflective stage of “low-risk” identity play (Parker, 2016, p. 427) to the “high-risk” stage of actively engaging with activities related to making a change, which will involve loss and may lead to rejection or failure, can be a significant stumbling block. April admitted: “*I love conversations about possibility. The challenge I find is moving on from that, so making a choice. I hold on to possibility for quite a long time.”* At this point, participants benefited from reminders of their motivations, and found it useful to set themselves step-by-step, achievable tasks involving the testing and exploring of “provisional selves” and potential futures (Ibarra et al., 2010). By achieving these tasks, they increased their sense of agency and raised their levels of confidence and self-efficacy (Leimon et al., 2011). Active reflection on the results from testing and experimenting was key to narrowing down “provisional selves” (Ibarra et al., 2010).

This challenging stage of the process often led to participants pausing the process, citing reasons such as training, children, location, money, or health. Targeted relational support (Motulsky, 2010), as well as more solution-focused approaches such as goal setting and follow-up, structure and strategy, played a significant role in making a decision and continuing with the process of career transition.

**The fourth stage, “How can I make this work—and still be me?**”, follows decision-making. This stage is about navigating the co-existence of old and future identities, absorbing the new landscape, learning about the environment, resources, people, expectations, figuring out the desired impact and contribution—while working toward the ultimate goal of developing and integrating new identities and “being me” in the new role (Sinclair, 2011). For some, such as Aria, it was about stripping away inauthentic layers: *I’ve been a fake version of myself that my employer wanted me to be, or I thought he did, and now I can shed that skin and be the real me, that’s always been there, I can be more authentic and honest*.

As participants began moving into this next phase of their lives, emotions were heightened. Fear and excitement ran hand in hand (Kidd, 2008). An initial period of discomfort in the new situation, a lesser episode of liminality during which the participants mourned the old and learned, adapted and grew into their new role, was recognized as the uncomfortable period necessary to actively craft a new working identity. This opportunity to learn and develop inspired women initially, but after an initial honeymoon period challenges not previously predicted arose, at a time when few if any support systems were in place. The need to change to meet new demands, letting go of familiar approaches, behaviors, skills and contacts in search of new ones, was found to be a daunting and solitary experience (Bonneywell, 2016; McGill et al., 2019). Andrea described her feelings in her new role: *Losing your sense of self, you feel like the things you were anchored to for your psychological safety, you are now mostly cut adrift from … I am in a hiatus, which is frightening sometimes.* Minor consequential transitions arising from the career change, or adjustment of attitude toward an existing role, often extended beyond the role and impacted others in the household (Fouad and Bynner, 2008), leading Cynthia and others to reflect that “*identity work is never ending*”. Cut loose from evidence of previous success and often feeling alone and challenged, they focused on their future self, and the identity work needed to become who they wanted to be. Looking inwards to past patterns of emotion, behavior and attitude in transitions (Sinclair, 2013), and looking outwards at role models, resources and support systems, were helpful activities for this group of women.

This study found that participants were sometimes prepared to delay personal expectations of authenticity while they experimented with and adapted to the new situation. Rather than being considered a sacrifice (De-Valle, 2014), this was seen as a pragmatic coping strategy. As in the previous stage, the safe space provided by coaching was perceived as valuable in supporting clients through the identity work of exploring and integrating emerging identities (McGill et al., 2019).

## The Me-As-a-Process coaching model

In his introduction to the Conceptual Encounter methodology, De Rivera invites the reader to “join with us in mapping personal experience” (de Rivera and Kreilkamp, 2006, p. 1). It therefore seemed appropriate to call the coaching model resulting from this CE study “The MAP (Me-As-a-Process) coaching model: women’s identity work in career choices and transitions.”

**Figure 1:**
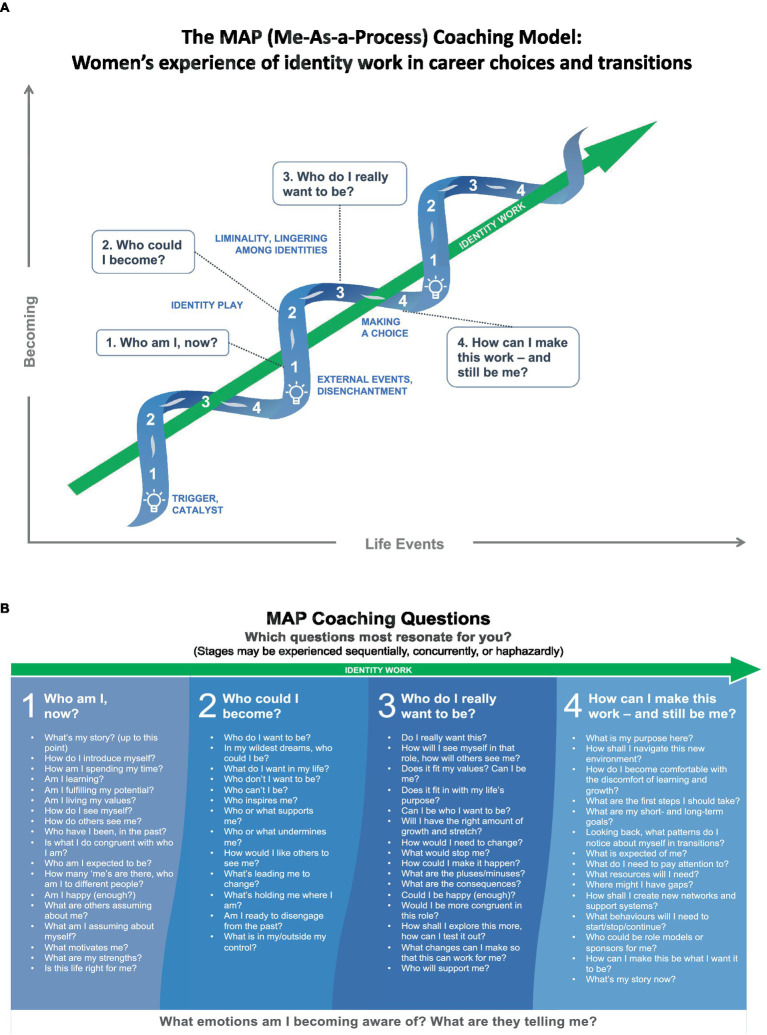
The MAP coaching model: women’s experience of identity work in voluntary career choices and transitions.

The MAP coaching model comprises two elements (Figures 1A,B, below). The first is an integrative perspective on the process of the identity work in intentional career change. This is represented by a spiral diagram describing the four stages that emerged from the research: “Who am I, now?”; “Who could I become?”; “Who do I really want to be?”; and “How can I make this work—and still be me?” These stages are shown as discrete, but in practice they interweave and are experienced haphazardly rather than sequentially, and often concurrently. While participants’ experiences of identity work in career choices and transition were unique and idiosyncratic, the four stages were discernible. The MAP spiral diagram (Figure 1A) builds upon Ibarra’s (2003) work, adding a preliminary stage “Who am I now?” for exploring identity and increasing self-awareness, in line with Peltier’s (2001, p. 185) assertion that in executive coaching, “awareness is everything.” In addition, rather than Ibarra’s single recurring cycle, it incorporates ongoing cycles of an iterative experience. This spiral design was a result of influences from Prochaska et al. (1992), whose work showed that people build on earlier experiences, and Ibarra and Barbulescu’s (2010) work on the link in identity work between past, present and future. Research participants specifically referenced the value of learning from earlier positive and negative experiences and building identity not as a one-off achievement but as an ongoing process (Watson, 2008; Brown, 2015, 2017).

The second element of the MAP model (Figure 1B) is the accompanying coaching questions. Whitmore (1992, p. 46) notes that his key criteria for successful coaching, awareness and responsibility, “are better raised by asking than by telling.” There are four sets of coaching questions, one for each of the model’s stages. The questions can be linked to the fundamental and holistic enquiry lying at the heart of career practice (Yates, 2017a): “How can I become who I want to be?” It’s a complex issue, requiring exploration of areas such as identity, transition and life-and work-design. Research partners who had been struggling with the experience of identity work in career transitions at a subconscious level found the questions helpful in bringing it to consciousness (Bourne and Özbilgin, 2008). One of them, Anita, commented: *Without actually being able to articulate them [the questions to reflect upon], it’s much harder.* The purpose of this second element of the MAP model is to stimulate awareness and personal, agentic responsibility through discussion and exploration.

Together, the two parts of the MAP model reflect the need for both insight and action (Prochaska et al., 1992) in the many transitions that are likely to form part of the lifelong process of “Becoming.”

## Implications for coaching practice

As coaching works with the self and is person-centered, it is ideal for facilitating conversations about identity work in transitions (Savickas, 2013; Yates, 2017a,b). Coaching can foster a psychologically safe space (Edmondson and Lei, 2014), referred to by Florent-Treacy (2009) and others as incubators or identity workspaces, which provide a secure base for exploring new beginnings. Often, coaching clients have conflicting desires and seek to explore the possibility of change away from vested interests and influencers. Within that confidential, safe space, I have found that coaching clients relish the opportunity to reflect on aspects of their identity, to investigate concepts such as identity, identity work, transitions, and to explore “unaskable” questions in a holistic approach to their lives, personal and professional (Adler, 2006, p. 239). Clients find intrigue and empowerment in understanding and naming their own “identity work”—the process of actively shaping and crafting who they want to be.

Coaching education traditionally teaches that “telling” closes down opportunities for growth in a client, while questioning and listening are seen as key to promoting growth, personal development and improved performance (Schein, 2013; Parker, 2016). While I strongly endorse this, I also agree with Kline (1999, p. 83) that sometimes “Accurate, complete information is vital if people are going to think for themselves, clearly and boldly.” For example, in my experience, simply sharing that career transition has been recognized as a psychological phenomenon can be positive for a client. Brief descriptions of contemporary views of “identity,” “careers,” and “transitions” contextualize their own experience and make them feel less alone. Validating transition as an established process that involves identity, and explaining that it is normal to find the experience challenging, lengthy, complex, and ultimately unique, reassures the client that they are not “uniquely unable” to manage it. Being shown a model representing the essential structure of the process was similarly valuable. Many of the women spoke about the relief of recognition on seeing the model and of the sense of organization and control provided by a framework (de Rivera and Kreilkamp, 2006). They appreciated it as a starting point for a discussion, a language with which to tackle an often hitherto unarticulated process (Bourne and Özbilgin, 2008).

For some, the experience of identity work in career choices and transitions continues for many years, often including a period of “time out.” The process is subjective and unique and can be major or minor, lengthy or short, easy or hard, and result in a change or not. The magnitude of the experience relates more to the perspective of the client, and the significance they give it (Leimon et al., 2011). Not every career choice is a struggle, and not everyone needs coaching to reach a resolution. The amount of coaching time dedicated to the various stages in the process of identity work in career transitions, and the areas for exploration, should be directed by the client. Effective coaching begins by establishing where the client is in the process, and starting work with them there (Prochaska et al., 1992; Leimon et al., 2011).

Executive coaching for women should not be gender neutral (Sinclair, 2013; Skinner, 2014; O’Neil et al., 2015). It needs to take into consideration the complex gendered conditions in which women work and address women’s specific situations and developmental needs. “Gender is a central aspect of our identity” (Peltier, 2001, p. 206), and for many of the participants, gender was at the heart of their experiences of identity work in career transitions. However, coaches should also be aware that gender does not play a significant role in transition for everyone (LaPointe, 2013).

Almost all the women experienced volatile emotions throughout the process (Appendix 8), aligning with Kidd’s (2008, p. 181) finding that “Emotions are fundamental to careers.” For the 53 women interviewed, it was not possible to determine a pattern to this emotional journey.

If the experience and process of identity work among the research partners was idiosyncratic, the goal was often similar. Research partners wanted to craft a congruent and dynamically evolving life with purpose and meaning, challenge, learning, control, status and balance. They also wanted to work actively toward aligning their career and their identity (Kira and Balkin, 2014), bringing more of themselves to their occupation and “being me” (Sinclair, 2011), rather than feeling pressured to adopt inauthentic (often masculine) behaviors. Career and work has to fit into women’s lives, rather than women fitting themselves into jobs (Savickas, 2008). Some clients experiencing the early stages of career transition may decide to stay where they are for the time being, perhaps remaining in a less demanding role in order to fulfill their personal sense of motherhood, or in an existing role for other pragmatic reasons. Coaching can act as an enabler (Skinner, 2014), freeing women to discover their own identity motives, clarify their current values and purpose, consider the choices available and holistically design their life (LaPointe, 2013). Whatever they choose, the decision is a conscious choice, putting them in charge. If they decide to stay where they are for the time being, that sense of empowerment gives them the energy to reframe their attitude to their current occupation and perhaps make changes to make it more purposeful.

## Using the MAP coaching model: suggestions for coaching practice

The process of identity work in career choices and transitions is not a single-session coaching topic, nor need it be the sole focus of a session—as it is ongoing, it can be revisited over the duration of a coaching program. Coaching identity work in career transitions explores both internal and external worlds, and their intersection (Parker, 2016). It involves discussions about identity. Every person’s experience of the process is unique, as is every coach’s approach; consequently there is no one coaching solution or prescribed technique (Leimon et al., 2011). Coaches have to be aware of, and respectful of, the potential consequences of uncovering things that might not have been previously seen or understood (O’Neil et al., 2015), and be “more thoughtful and appropriately tentative” in discussions about identity (Bachkirova, 2020, p. 2). Given the complex, challenging and emotional nature of this coaching work (Terblanche et al., 2018), coaches should have regular supervision (Cox and Bachkirova, 2007).

Coaches should be led by their client in their use of the MAP model. It may be useful to introduce it all at once to the client, or it might be more beneficial to bring in individual aspects of it as appropriate. Coaching clients value the model for its effect of normalizing their experience, providing a language to discuss and explore it, and providing a marker to indicate where they might be in the experience.

Clients naturally tend toward a narrative framing of who they were, and storytelling provides an appealing and revisable approach to finding meaning, purpose and direction (Pemberton, 2015). The stories we tell about ourselves and the world around us help us understand who we want to be, and provide a constructive approach to navigating identity opportunities, threats and reconstruction, and our unique response to each of those (Yip et al., 2020). At each stage of coaching the process of identity work in career transitions, the use of narrative allows clients to explore patterns, reveal possibly outdated assumptions and expectations, come to terms with endings and loss, and craft new versions of themselves. The entire set of coaching questions is framed by using narrative as a coaching tool, with the first and last questions referencing it.

Clients, who often find the experience of career transition isolating and challenging (De-Valle, 2014; McGill et al., 2019), work best with a coaching style that is holistic and agile, able to hold contradictions and ambiguity (Leimon et al., 2011). The element of positive regard at the heart of coaching identity work in career transitions is key, and objective, constructive challenge is also valued (Motulsky, 2010; Weinstock, 2011; De-Valle, 2014).

Below is a summary of the type of exercises I have found most valuable in coaching sessions. However, as we have seen, there is no template and each coaching commission should be approached as a unique situation. The suggestions are summarized in the table below.

**Stage 1: Who am I now?**, begins with an assessment of the current situation including levels of happiness, fulfillment, resilience, health, use of potential, challenge and stretch. Discussion of past and present influences on identity—“how did I get here?”—increases levels of self-awareness and personal responsibility for attitudes and behaviors. Kline’s (1999) “Limiting Assumptions” encourage individuals to reflect on their assumptions, questioning and reframing them to achieve personal transformation (Pinkavova, 2010). Life timelines, values exercises, career-happiness graphs, revisiting earlier transitions and strengths work all provide perspective and can help create an internal compass to guide future career choices. Once introduced to the agentic concept of “life-design,” clients find the idea of actively planning how to become who they wanted to be exhilarating and empowering (Savickas et al., 2009; Yates, 2017a).

In **Stage 2, Who could I become?**, clients benefit from the confidential and accepting space of coaching that gives them permission to explore and articulate their possible and impossible selves, and thereby establish identity motives. Coaches will need to support them in holding simultaneous, ambiguous and inconsistent truths. This stage encourages clients to dream and be creative, so free writing or letters to future selves, visual aids or drawing, envisioning exercises, painting or other creative pursuits may be useful. Identifying inspirational role models is valuable.

**Stage 3, Who do I really want to be?**, calls for the coach to provide targeted support and encouragement as clients face up to exposing their dreams and ideas to reality. Helpful interventions at this point include solution-focused approaches, chunking down, goal setting, structured activity and follow-up. Mapping of contacts and networks can create a plan for reaching out and connecting. In some cases, women want support with practical skills such as writing a c.v., and since this is usually outside the scope of executive coaching, coaches should be prepared to signpost relevant resources. Coaching to boost emotional, physical and mental resilience will be key. A decision to pause the process can be contextualized as “normal,” just part of the process of transition.

**Stage 4, How can I make this work—and still be me?** is often where clients feel they are when their executive coaches meet them. Instead of the anticipated positive feelings associated with decision-making, clients are often embarrassed at feeling challenged by the new cycle of liminality, not-knowing and adaptation as they navigate their way through the new landscape and its systems. Contextualizing this as normal, and part of the process of transition, is itself valuable to clients. It is useful to talk through earlier stages of identity work with the client so they can see how they arrived at this point; revisiting life purpose and values, and encouraging experimentation with behaviors in those lights, can be positive steps toward finding the path to “becoming me” in a new situation. Analyzing patterns of experience during previous transitions is helpful, reminding participants that they had previously overcome difficulties. Assessing what undermined them then, and what helped them to succeed, and applying the learning to their current situation, increases confidence (McGill et al., 2019). Seeing the period of transition in the context of a whole-life timeline can also help with perspective and encourage resilience (Pemberton, 2015). Evaluating the strengths that got them here and surveying how to use them in the new situation reinforces their ability to meet the challenge. Support to address purpose, vision and strategy, often perceived by organizations as an area in which women lack confidence and ability (Ibarra and Obodaru, 2009), helps to create valuable milestones and pathways. Internal and external resources for achieving a successful transition can be explored and identified. Role models are valuable in identity construction, but as senior female women are scarce in many organizations, a less stereotypical attitude toward gender in role models should be adopted (Sealy and Singh, 2009). Dweck’s (2006) growth mindset “not yet” is a valuable coaching approach at this stage (Parker, 2016). Finally, clients can be supported in reshaping, reframing and amending their narratives, to provide cohesion, shape and sense through language (Savickas, 2008; Pemberton, 2015; Yates et al., 2017; Bachkirova, 2020). As Willig (2001) suggests, it is not events that make us who we are; what is decisive is the process by which we assimilate the unfamiliar, resolve the contradictions and conflict, and construct a particular version of ourselves.

**Table 1:** 

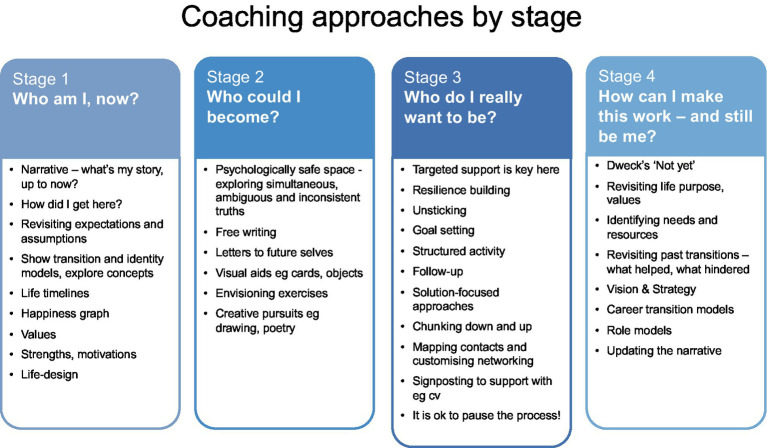

## Conclusions and further research

The significance of this research lies in its focus on women’s identity work as they face career choices and transitions, and in its development of a coaching model, designed to help coaches support their clients effectively during this process. The model is a co-creation, actively crafted by the researcher and research partners, all of whom had recent experience of the topic. Its primary purpose is to encourage conscious engagement with and agency in the process of holistic identity work, rather than to develop women leaders, or even to successfully complete a career transition (although I would be delighted if these were secondary consequences). It is not intended to be predictive or prescriptive, but exploratory and tentative.

Brown (2017) reminds us of the need to continually re-evaluate what we think we know about identity, in order to mitigate complacency and provoke novel insights. Going forward, the model can be subject to ongoing revision as more input is received from broader groups of participants. Each of the concepts forming part of this process—identity and identity work, gender, transition and careers—are rich areas for ongoing research within the coaching and mentoring field. Widening the criteria for participation to include more socially representative groups would offer valuable insights. Additionally, more research is needed into the role of emotions in this process, “to understand the mutual constitution of emotions and identity work” (Winkler, 2018, p. 129). New insights might also be provided by longitudinal, real-time research using methodologies such as heuristic or Action Research, rather than retrospective accounts, or a Grounded Theory approach, allowing theory to emerge without an initial conceptualization.

Given the lack of education in the coaching field about the process of identity work in career choices and transitions (Parker, 2016; Caza et al., 2018), more research could lead to the development of a coach-specific theory for inclusion in executive coach education and training. In 2009–12, when I studied for a Masters in Coaching and Behavioral Change, there was little or no reference to concepts such as transition, identity and identity work, or the changing nature of careers. I argue that in order to effectively support clients with this process, coaches will benefit from learning about these concepts. Increasingly there is recognition that a wider, multidisciplinary theoretical base to coaching education would benefit the profession (Griffiths and Campbell, 2008; Yates et al., 2017).

This article supports an identity-based approach to coaching career choices and transitions. The research is timely. With the rise in disruptive change faced in all areas of our society and organizations, including the global lockdown as a result of the Covid-19 pandemic, individuals will benefit from consciously engaging in the lifelong project of constructing a self (Savickas, 2013). The MAP coaching model will bring the topics of identity and identity work into the coaching arena, supporting coaches and clients as they navigate career transitions and dynamically engage in becoming who they want to be.

## Data availability statement

The raw data supporting the conclusions of this article will be made available by the authors, without undue reservation.

## Ethics statement

The studies involving humans were approved by Oxford Brookes University Ethics Committee. The studies were conducted in accordance with the local legislation and institutional requirements. The participants provided their written informed consent to participate in this study. Written informed consent was obtained from the individual(s) for the publication of any potentially identifiable images or data included in this article.

## Author contributions

SS: Writing – original draft.
